# A transgenic minipig model of Huntington's disease shows early signs of behavioral and molecular pathologies

**DOI:** 10.1242/dmm.035949

**Published:** 2018-10-24

**Authors:** Georgina Askeland, Marie Rodinova, Hana Štufková, Zaneta Dosoudilova, Monika Baxa, Petra Smatlikova, Bozena Bohuslavova, Jiri Klempir, The Duong Nguyen, Anna Kuśnierczyk, Magnar Bjørås, Arne Klungland, Hana Hansikova, Zdenka Ellederova, Lars Eide

**Affiliations:** 1 Department of Medical Biochemistry, Institute of Clinical Medicine, University of Oslo, 0372 Oslo, Norway; 2 Department of Microbiology, Oslo University Hospital, 0372 Oslo, Norway; 3 Department of Pediatrics and Adolescent Medicine, First Faculty of Medicine, Charles University in Prague and General University Hospital in Prague, Prague 12808, Czech Republic; 4 Laboratory of Cell Regeneration and Plasticity, Institute of Animal Physiology and Genetics, Czech Academy of Science, Libechov 27721, Czech Republic; 5 Department of Cell Biology, Faculty of Science, Charles University in Prague, Prague 12843, Czech Republic; 6 Department of Neurology and Centre of Clinical Neuroscience, First Faculty of Medicine, Charles University in Prague and General University Hospital in Prague, Prague 12821, Czech Republic; 7 Proteomics and Metabolomics Core Facility, PROMEC, Department of Clinical and Molecular Medicine, Norwegian University of Science and Technology, 7491 Trondheim, Norway

**Keywords:** Huntington's disease, Mitochondrial function, DNA damage, DNA repair, HD large animal model

## Abstract

Huntington's disease (HD) is a monogenic, progressive, neurodegenerative disorder with currently no available treatment. The Libechov transgenic minipig model for HD (TgHD) displays neuroanatomical similarities to humans and exhibits slow disease progression, and is therefore more powerful than available mouse models for the development of therapy. The phenotypic characterization of this model is still ongoing, and it is essential to validate biomarkers to monitor disease progression and intervention. In this study, the behavioral phenotype (cognitive, motor and behavior) of the TgHD model was assessed, along with biomarkers for mitochondrial capacity, oxidative stress, DNA integrity and DNA repair at different ages (24, 36 and 48 months), and compared with age-matched controls. The TgHD minipigs showed progressive accumulation of the mutant huntingtin (mHTT) fragment in brain tissue and exhibited locomotor functional decline at 48 months. Interestingly, this neuropathology progressed without any significant age-dependent changes in any of the other biomarkers assessed. Rather, we observed genotype-specific effects on mitochondrial DNA (mtDNA) damage, mtDNA copy number, 8-oxoguanine DNA glycosylase activity and global level of the epigenetic marker 5-methylcytosine that we believe is indicative of a metabolic alteration that manifests in progressive neuropathology. Peripheral blood mononuclear cells (PBMCs) were relatively spared in the TgHD minipig, probably due to the lack of detectable mHTT. Our data demonstrate that neuropathology in the TgHD model has an age of onset of 48 months, and that oxidative damage and electron transport chain impairment represent later states of the disease that are not optimal for assessing interventions.

This article has an associated First Person interview with the first author of the paper.

## Abstract (precis)

**Summary:** Here, we show that a minipig model of Huntington's disease mimics human neurodegeneration and holds promise for future intervention studies. However, minipig peripheral blood mononuclear cells express no detectable mutant huntingtin, eliminating their use as monitoring tools.

## INTRODUCTION

Huntington's disease (HD) is a devastating neurodegenerative disease for which there are currently no disease-modifying treatments. HD is inherited in an autosomal-dominant manner and caused by a trinucleotide CAG expansion in exon 1 of the huntingtin gene (*HTT*), resulting in the expression of mutant huntingtin (mHTT). It is clinically characterized by cognitive, psychiatric and motor dysfunctions, along with weight loss and muscle wasting. These symptoms stem from the characteristic striatal neurodegeneration, thought to be the neuropathological hallmark of the disease. The CAG repeat size negatively correlates with the age of onset of the disease and explains most of the variation in age at motor onset, with the remaining variance being currently unidentified genetic and environmental factors.

The *HTT* gene is essential for viability, and the CAG expansion mutation might cause neurodegeneration via a toxic effect of the mHTT protein, reduction of endogenous HTT function or a combination of both. Fragments of mHTT are identified in both patient brains and HD rodent models (Davies et al., 1997; DiFiglia et al., 1997) and fragmentation correlates with disease progression (Mende-Mueller et al., 2001). The fragments can be produced by proteolytic cleavage of the full-length mHTT or alternative splicing (Miller et al., 2010; Sathasivam et al., 2013).

A genetic hallmark of expanded CAG repeats is that they are able to spontaneously form hairpin structures that act as intermediate structures in somatic expansions. In mouse models, it has been demonstrated that faulty DNA repair catalyzes age-correlated CAG mutagenesis in a tissue-dependent manner (Jonson et al., 2013; Kovtun et al., 2007; Mollersen et al., 2012). Preliminary data from a mouse model of HD indicate that the somatic mutagenesis actually contributes to the neurodegeneration, possibly in a crosstalk mechanism between mismatch DNA repair and base excision DNA repair (Lai et al., 2016; Pinto et al., 2013).

The underlying mechanistic cause of HD progression by mHTT is still under investigation. As indicated above, DNA damage and repair is involved in somatic mutagenesis, and some studies have identified elevated levels of the oxidative DNA damage marker 8-oxoguanine (8-oxoG) in HD (Bogdanov et al., 2001; Long et al., 2012; Polidori et al., 1999), although we (Askeland et al., 2018) and others (Borowsky et al., 2013) could not confirm these findings in peripheral blood mononuclear cells (PBMCs) from HD patients. It has been proposed that mHTT directly inhibits mitochondrial function by inducing oxidative stress and correlates with increasing CAG length (Hands et al., 2011). Postmortem striatum samples from advanced-stage HD patients showed reduced activity of complexes II, III and IV (Browne et al., 1997; Gu et al., 1996), while the R6/2 HD mouse model has shown reduced aconitase activity in the striatum and reduced complex IV activities in the striatum and cerebral cortex (Tabrizi et al., 2000). In a recent study, we discovered signs of reduced mitochondrial activity in PBMCs from HD patients, despite normal biochemical complex activities (Askeland et al., 2018).

Mouse models of HD, like the R6/2 model, have been used to test compounds that might be neuroprotective, such as the mitochondrial coenzyme Q10 (coQ10), especially in combination with remacemide (Ferrante et al., 2002). However, in follow-up clinical testing in HD patients, coQ10 failed to show a significant slowing of functional decline in early-stage HD patients, even at very high doses (Huntington Study Group, 2001; McGarry et al., 2017). Similarly, clinical trials for the use of creatine in early-stage HD patients also failed (Hersch et al., 2017), even though striking results were seen in the preclinical studies in R6/2 mice (Dedeoglu et al., 2003; Ferrante et al., 2000). This raises an important question of whether preclinical testing in current HD animal models can accurately reflect efficacy in humans. In addition, current biomarkers used in clinical trials are suboptimal owing to variability (Unified Huntington's Disease Rating Scale) or long response time MRI, and there is a particular need for reliable markers for pre-manifest HD. To accelerate the discovery of effective treatments for HD, a two-pronged approach might be needed, developing both better models and more accurate biomarkers.

In 2013, the Libechov transgenic minipig model for HD (TgHD) was first described (Baxa et al., 2013). A lentiviral approach was used to insert a truncated N-terminal fragment of exon 1 of human mHTT (1-548 amino acids). This was under the control of the human *HTT* promotor and contained 124 glutamines (CAGCAA repeats). This was the first transgenic HD pig model with successful germline transmission. The phenotype of the TgHD model is apparently mild. No aggregate formation is seen in brain tissue up to 16 months, and developmental or motor deficits are absent up to an age of 40 months (Baxa et al., 2013). However, levels of DARP32, a marker of medium spiny neurons (Gustafson et al., 1992), were reduced in the neostriatum at an age of 16 months. At 13 months, TgHD minipigs exhibited reduced fertility and lower sperm count, implying that a HD phenotype manifests prior to motor deficits. Characterization of the model is ongoing, as the original and subsequent generations of TgHD minipigs age, to provide a detailed description of the model. A battery of novel tests has recently been developed to assess the phenotype of the TgHD minipig model. These include a gait test, a hurdle test and a startbox back-and-forth test (Schramke et al., 2016). Effective preclinical testing in this model would be greatly promoted by a quantifiable and reliable phenotype.

We postulate that the TgHD pig model is a good representation of the human HD pathology, and that molecular parameters in this model are likely candidates to monitor disease progression and therapeutic interventions in humans. In order to test this hypothesis, we applied a repertoire of molecular and functional analyses to correlate with the HD phenotype and determined the age of onset.

## RESULTS

### The TgHD minipig model recapitulates behavioral abnormalities consistent with HD

**Fig. 1.**
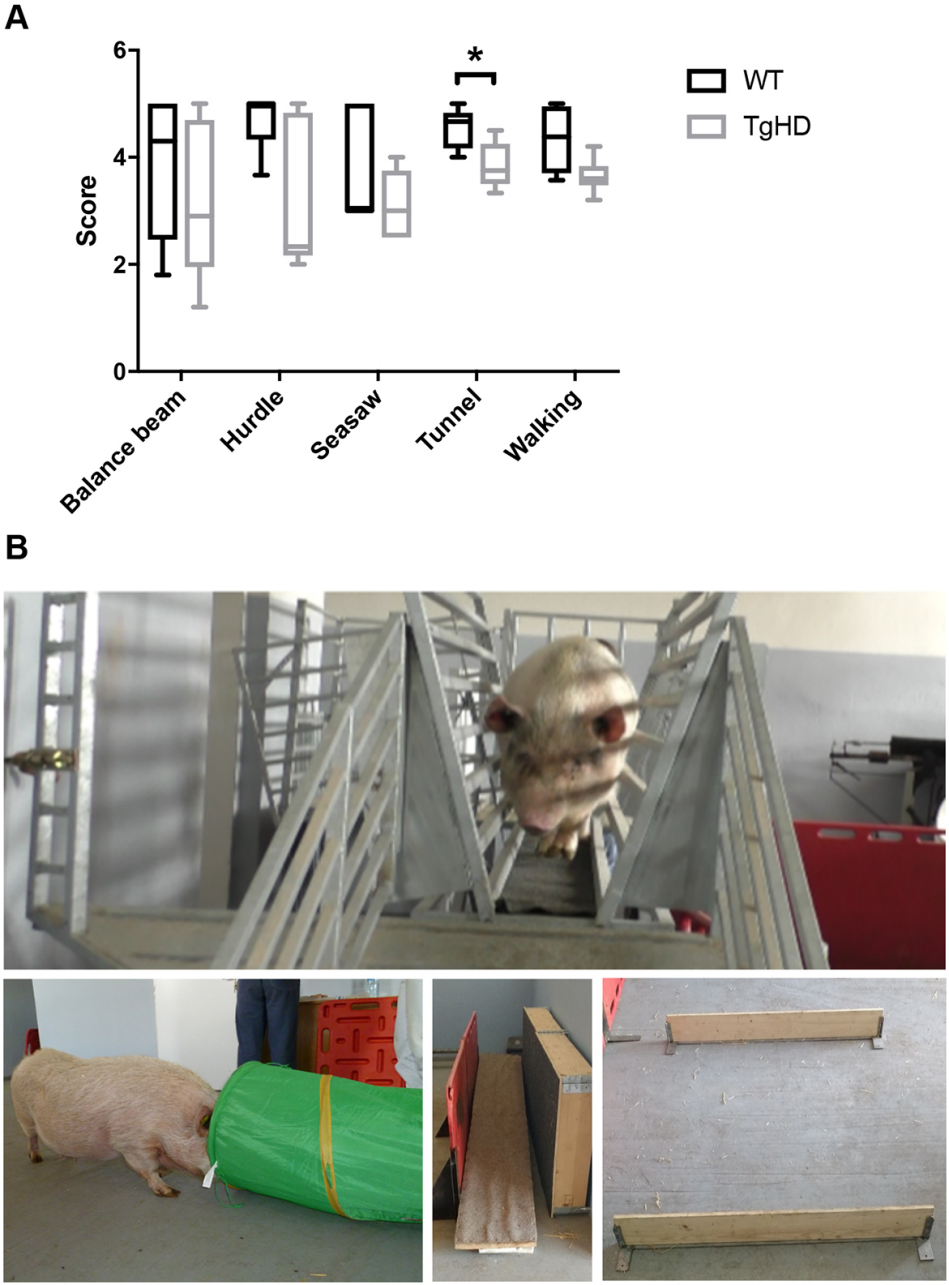
**The TgHD minipig model shows behavioral abnormalities consistent with early neurodegeneration.** (A) Behavioral testing with tests specially designed for minipigs revealed a deficit in performing the tunnel test (*P*=0.04) and indications of a deficit in walking (nonsignificant). (B) Representative images (clockwise from top) of the balance beam, hurdle, seesaw and tunnel tests. Mann–Whitney test and two-way ANOVA, *P<0.05. Box plots and whiskers indicate minimum to maximum values, with hinges representing the 25th and 75th percentiles and the median indicated by the centerline. Sample size [female (F)+male (M)] distribution: balance beam, WT 4F+2M, TgHD 4F+2M; hurdle, WT 3F+2M, TgHD 4F+1M; seesaw, WT 3F+2M, TgHD 3F+2M; tunnel, WT 3F+2M, TgHD 4F+1M; walking, WT 4F+2M, TgHD 4F+2M.

A battery of novel behavioral tests designed for minipigs has been established (Schramke et al., 2016). Stance, gait and ability to cross a barrier were normal in F0 and F1 generations of TgHD minipigs up to 40 months of age, although fertility was affected (Baxa et al., 2013; Schuldenzucker et al., 2017). We investigated motor performance, cognitive performance and/or behavior deficit in 48-month-old minipigs and detected a significant decline in the ability to perform the tunnel test in the TgHD minipigs (Fig. 1, *P*=0.04). There was also a general tendency for reduced performance in the other tests (nonsignificant). Thus, these results showed the onset of locomotor/neurological impairment in the TgHD minipig model at the age of 48 months.

### Genome integrity is affected in HD minipigs

**Fig. 2.**
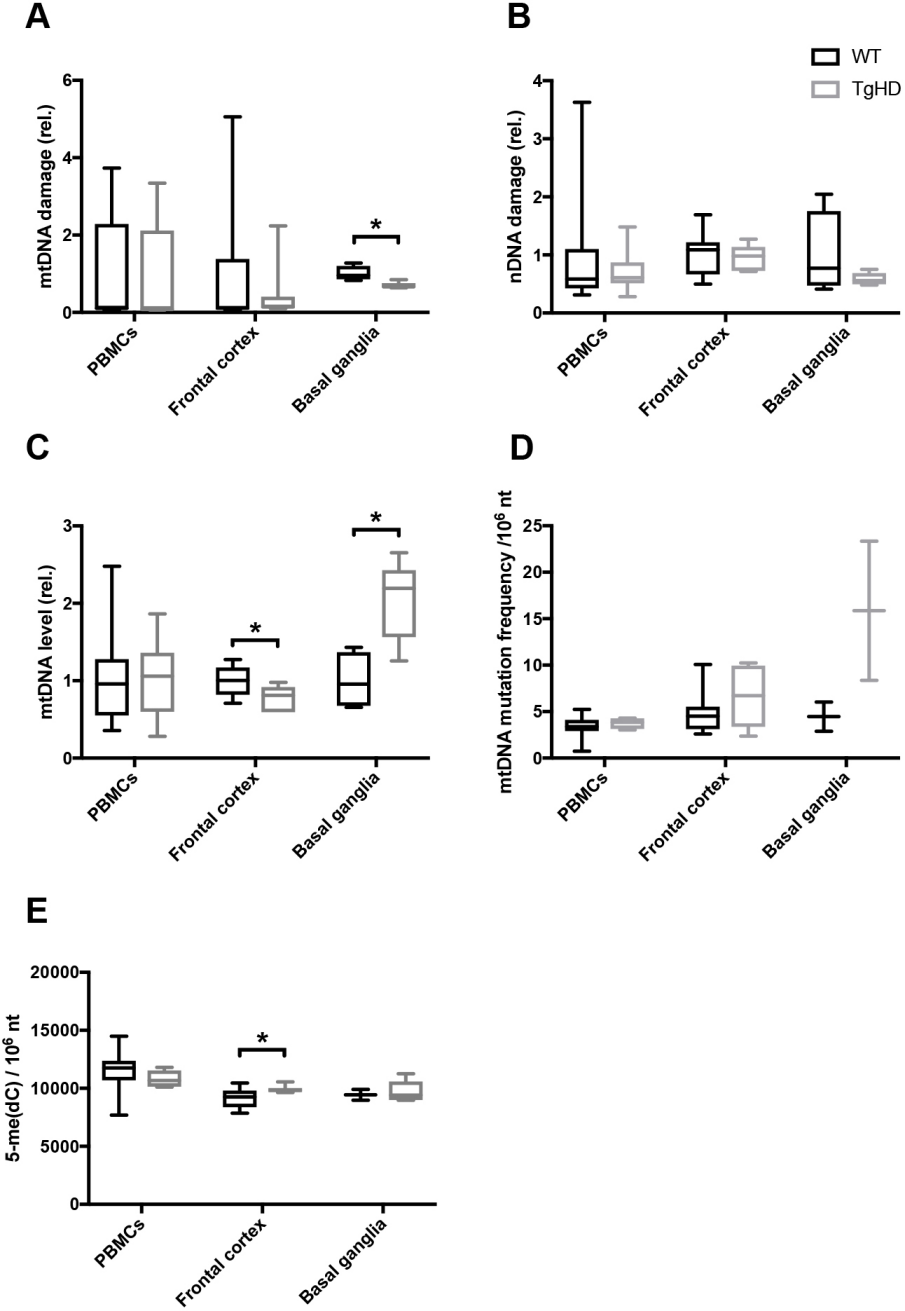
**Tissue-specific changes in genome integrity in the TgHD minipig model.** (A) mtDNA damage analysis revealed lower damage levels in the basal ganglia in TgHD than in WT (*P*=0.01), but no changes in PBMCs or the frontal cortex. (B) nDNA damage analysis demonstrated that nDNA integrity is apparently unaffected in the TgHD model. (C) Quantification of mtDNA copy number shows a significant decrease in the frontal cortex (*P*=0.04) and, in contrast, an increase in the basal ganglia (*P*=0.01) in the TgHD minipigs, relative to WT. No differences were seen in PBMCs. (D) mtDNA mutation frequency analysis indicates higher levels in the basal ganglia in TgHD (nonsignificant) than in WT, but normal levels in PBMCs and the frontal cortex. (E) The methylation mark 5-me(dC) was increased in the frontal cortex in TgHD compared with WT (*P*=0.047). No differences in 5-me(dC) levels were seen in PBMCs or the basal ganglia. Student's *t*-test, *P<0.05. Box plots and whiskers indicate minimum to maximum values, with hinges representing the 25th and 75th percentiles and the median indicated by the centerline. Sample sizes: (A) basal ganglia, WT *n*=4, TgHD *n*=5; PBMCs, WT *n*=17, TgHD *n*=14; frontal cortex, WT *n*=10, TgHD *n*=7; (B) PBMCs, WT *n*=17, TgHD *n*=14; frontal cortex, WT *n*=10, TgHD *n*=7; basal ganglia, WT *n*=4, TgHD *n*=5; (C) frontal cortex, WT *n*=10, TgHD *n*=5; basal ganglia, WT *n*=4, TgHD *n*=5; PBMCs, WT *n*=17, TgHD *n*=13; (D) basal ganglia, WT *n*=2, TgHD *n*=2; PBMCs, WT *n*=7, TgHD *n*=4; frontal cortex, WT *n*=10, TgHD *n*=6; (E) frontal cortex, WT *n*=10, TgHD *n*=7; PBMCs, WT *n*=10, TgHD *n*=6; basal ganglia, WT *n*=2, TgHD *n*=5.

We recently identified genome integrity as an early biomarker in peripheral PBMCs from HD patients (Askeland et al., 2018). The corresponding cells, in addition to brain regions from TgHD minipigs and their controls, were therefore assessed for DNA damage, mitochondrial copy number and DNA repair activity. Surprisingly, we did not identify any age-associated alterations in any of the parameters assessed (data not shown). In order to search for impact of the genotype, the different age groups were pooled for each genotype prior to reanalyses. The pooled samples revealed a reduced level of mitochondrial DNA (mtDNA) damage in the basal ganglia in TgHD minipigs, whereas the corresponding levels in the frontal cortex and in PBMCs were normal (Fig. 2A, *P*=0.01). Nuclear DNA (nDNA) damage was not significantly altered in any of the tested tissues in TgHD minipigs (Fig. 2B), thereby contrasting the situation seen in human PBMCs. We followed up on the level of mtDNA, and found lower mtDNA copy number in the frontal cortex in TgHD than in wild type (WT), but higher copy number in the basal ganglia in TgHD than in WT (Fig. 2C, *P*=0.04 and *P*=0.01, respectively). The differences in level and integrity of mtDNA in the basal ganglia might imply that mitophagy is disturbed in the basal ganglia of TgHD. We followed up on this point by assessing mtDNA mutation frequency. Although there was no indication of any altered mtDNA mutation frequency in PBMCs and cortex, there appeared to be a tendency to more mtDNA mutations in the basal ganglia in TgHD than in WT (Fig. 2D, nonsignificant), which could be an indication of ineffective mitophagy in the basal ganglia. 5-Methylcytosine [5-me(dC)] is a base modification that regulates gene regulation and global 5-me(dC) alters with differentiation, during development and with differences in aerobic activity. This epigenetic mark is found altered in untranslated regions in HD (Villar-Menéndez et al., 2013). When we assessed global 5-me(dC) levels in WT and TgHD minipigs, we found a modest, but significant, increase in the frontal cortex in TgHD minipigs (Fig. 2E, *P*=0.047), whereas PBMCs and basal ganglia displayed normal levels. 5-Hydroxymethylcytosine, another epigenetic marker, was not affected in TgHD minipigs (Fig. S2A). The different impact of mHTT on mtDNA copy number and mtDNA damage in the basal ganglia and cortex is indicative of the two brain regions being differentially affected. In contrast to the human situation, PBMCs were unaffected in the TgHD minipig.

### Tissue-specific variations in level of and DNA repair capacity towards 8-oxoG in HD minipigs

**Fig. 3.**
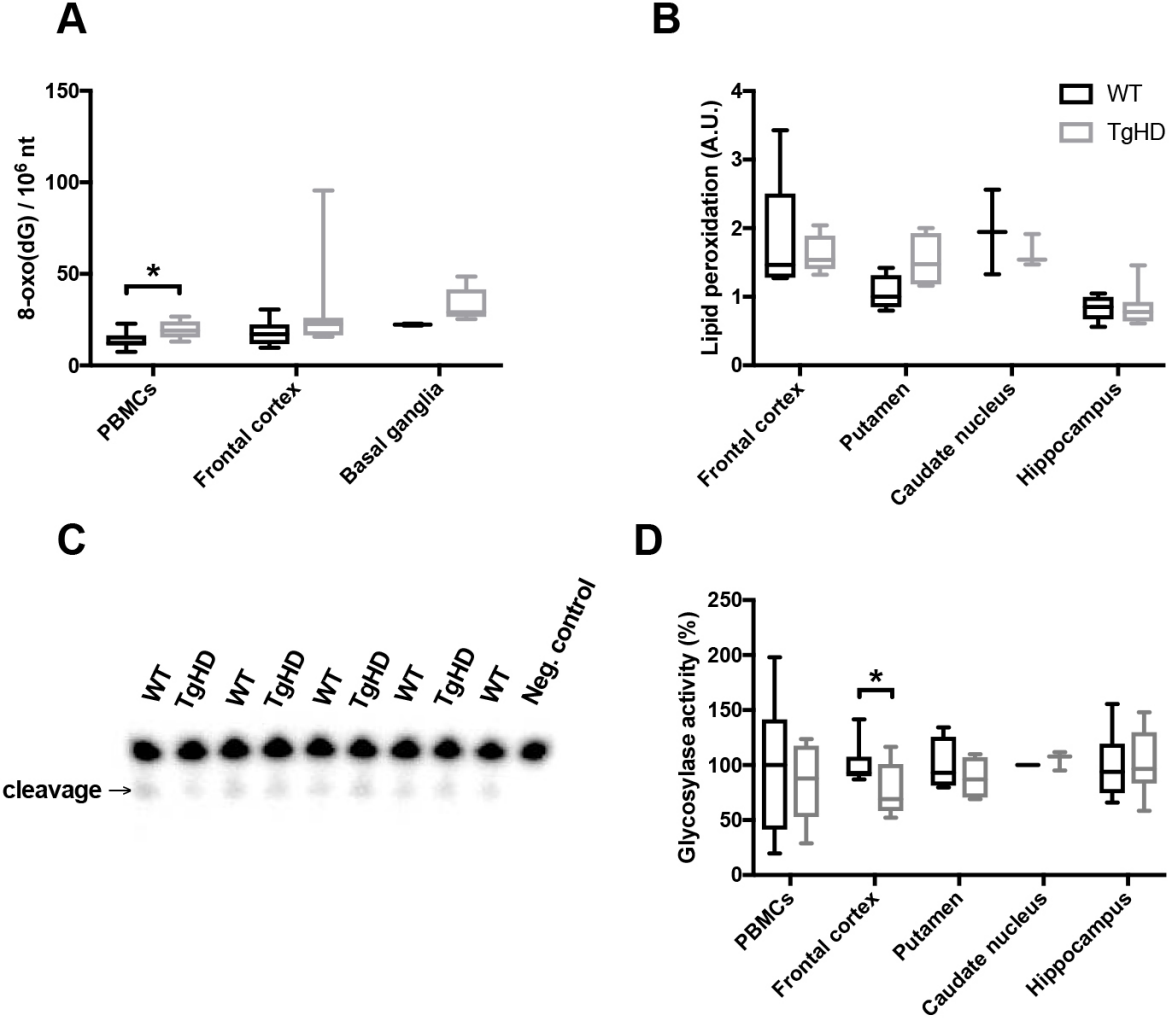
**Tissue-specific alterations in oxidative DNA damage and repair in TgHD minipigs.** (A) Mass spectrometry analysis of the oxidative damage marker 8-oxoG [as nucleoside 8-oxo(dG)] showed increased levels of PBMCs in TgHD relative to WT (*P*=0.03), whereas the frontal cortex and basal ganglia displayed similar levels in the two genotypes. (B) The level of malondialdehyde (MDA) was determined in the specified brain subregions. No significant differences were found between WT and TgHD in any of the groups. Arbitrary units (A.U.) represent levels of MDA (μg), showing the extent of lipid peroxidation. (C) Representative image of the DNA glycosylase activity assay, showing incision of 8-oxoG containing ^32^P-endlabeled oligonucleotide. Extracts were collected from individual animals. (D) DNA glycosylase activity toward 8-oxoG in nuclear protein extracts from PBMCs and different brain subregions. The analysis of substrate cleavage by DNA glycosylase enzyme OGG1 revealed reduced activity in the frontal cortex in TgHD (*P*=0.03), and indicates a defect in DNA repair of oxidative DNA damage. No genotype differences were seen in other brain regions. Data are presented as relative to the average of the WT activities. Student's *t*-test, *P<0.05. Box plots and whiskers indicate minimum to maximum values, with hinges representing the 25th and 75th percentiles and the median indicated by the centerline. Samples sizes: (A) PBMCs, WT *n*=10, TgHD *n*=6; frontal cortex, WT *n*=10, TgHD *n*=7; basal ganglia, WT *n*=2, TgHD *n*=5; (B) frontal cortex, WT *n*=9, TgHD *n*=9; putamen, WT *n*=4, TgHD *n*=4; caudate nucleus, WT *n*=2, TgHD *n*=3; hippocampus, WT *n*=7, TgHD *n*=7; (D) PBMCs, WT *n*=7, TgHD *n*=8; frontal cortex, WT *n*=9, TgHD *n*=10; putamen, WT *n*=4, TgHD *n*=4; caudate nucleus, WT *n*=2, TgHD *n*=3; hippocampus, WT *n*=7, TgHD *n*=8.

The reduction in mtDNA damage observed in the basal ganglia of TgHD could be ascribed to reduced mitochondrial activity and associated oxidative stress. To assess the status of oxidative stress in the TgHD, we first measured the level of 8-oxoG, a frequently used marker of oxidative stress. There was a tendency towards increased 8-oxoG levels in all tissues, but this change was statistically significant only in PBMCs (Fig. 3A, *P*=0.03). We validated the results by assessing levels of malondialdehyde (MDA), another marker of oxidative stress that is produced by lipid peroxidation. We tested several brain subregions, but found no significant differences in the levels of MDA (Fig. 3B) and conclude that the TgHD minipigs do not show evidence of oxidative stress in the brain up to 48 months of age. We followed up on the increased level of 8-oxoG in PBMCs by measuring the level of another oxidized base lesion, 5-hydroxycytosine. Interestingly, whereas 5-hydroxycytosine levels varied greatly between PBMCs and the brain subregions, there was no significant difference between WT and TgHD minipigs (Fig. S2B). The elevated level of 8-oxoG is therefore indicative of an altered repair activity towards this lesion in TgHD PBMCs. The main source of 8-oxoG removal is by 8-oxoG DNA glycosylase activity. We assessed the DNA glycosylase activity against 8-oxoG in cell-free extracts and found significantly reduced levels in the frontal cortex (Fig. 3D, *P*=0.03), whereas PBMCs, as well as other brain regions, exhibited normal DNA glycosylase activities.

### Unperturbed mitochondrial bioenergetics in HD minipigs

**Fig. 4.**
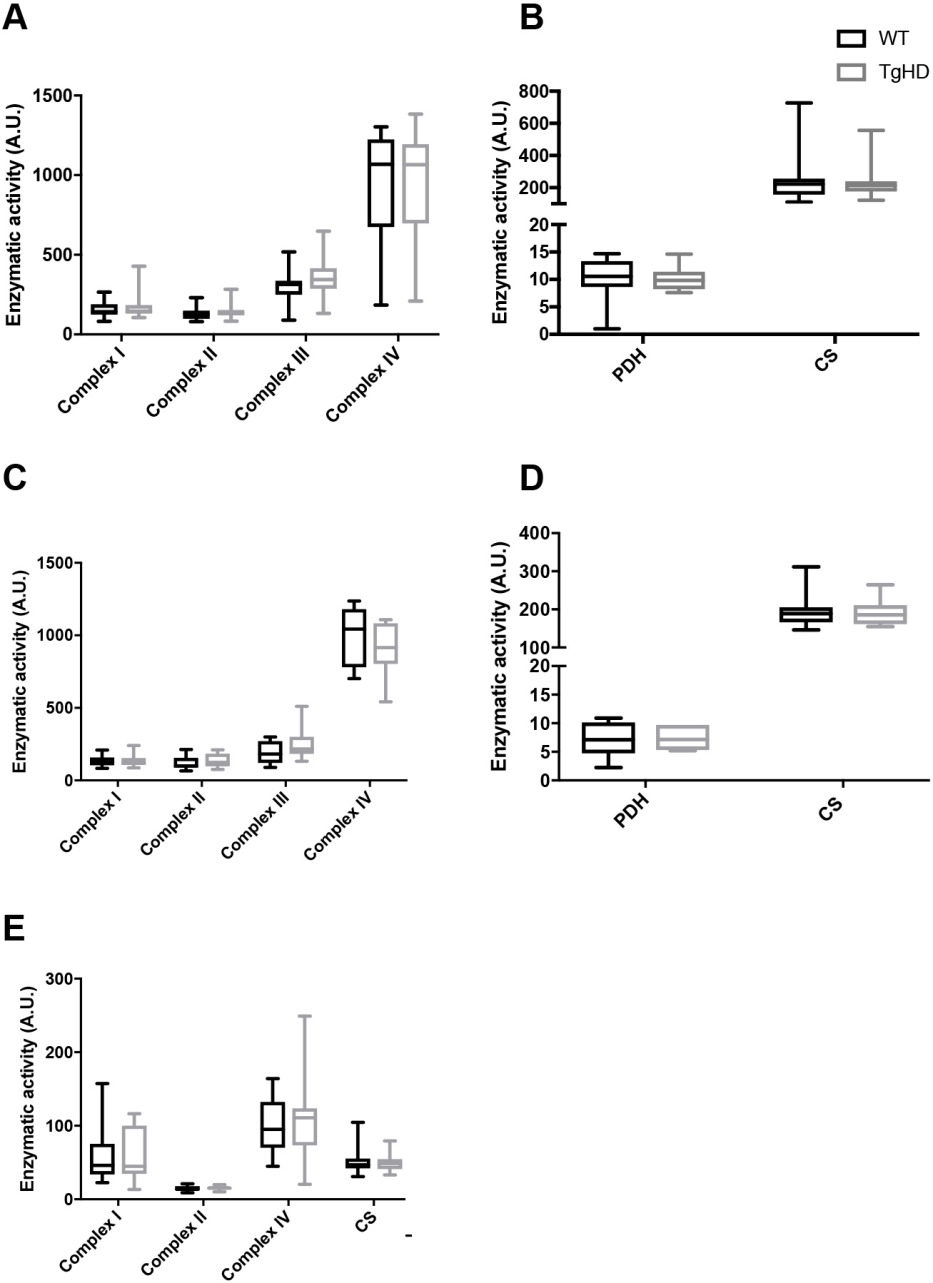
**Mitochondrial status in TgHD minipig brain subregions and PBMCs.** (A,B) Mitochondrial ETC complexes I-IV (A), pyruvate dehydrogenase (PDH) and citrate synthase (CS) (B) all showed normal activity in TgHD compared with WT in the frontal cortex. (C,D) In the basal ganglia, mitochondrial ETC complexes I-IV (C), PDH and CS (D) all showed normal activity in TgHD compared with WT. (E) In addition, mitochondrial ETC complexes I, II and IV and CS showed normal activity in TgHD compared with WT in PBMCs. Arbitrary units (A.U.) represent enzymatic activity in nmol/min/mg. Student's *t*-test was used to assess significance. Box plots and whiskers indicate minimum to maximum values, with hinges representing the 25th and 75th percentiles and the median indicated by the centerline. Sample sizes: (A,B) frontal cortex: complex I, WT *n*=19, TgHD *n*=20; complex II, III and IV, all WT *n*=19, TgHD *n*=21; PDH, WT *n*=16, TgHD *n*=17; CS, WT *n*=19, TgHD *n*=21; (C,D) basal ganglia: complex I-IV, all WT *n*=10, TgHD *n*=9; PDH, WT *n*=9, TgHD *n*=7; CS, WT *n*=10, TgHD *n*=9; (E) PBMCs: complex I, II and IV and CS, all WT *n*=18, TgHD *n*=19.

We recently identified mitochondrial aberrations in peripheral tissue from HD patients, including reduction of the subunit B of succinate dehydrogenase (SDHB) after onset of disease, which did not correlate with total functional score, an estimate for disease progression. To evaluate the putative impact of mitochondrial aberrations in the minipig model, we assessed mitochondrial parameters in TgHD and WT minipigs in two brain regions, and in PBMCs. First, pyruvate dehydrogenase (PDH) activity was unchanged in the frontal cortex and basal ganglia, indicating normal carbohydrate oxidation (Fig. 4B,D). Citrate synthase (CS) activity is a marker of mitochondrial volume, and was also similar between WT and TgHD minipigs in the two brain regions and PBMCs examined, indicating that mitochondrial capacity in the brain regions, as well as in PBMCs, is not affected in the TgHD model (Fig. 4B,D,E). These findings were consistent with the similar mitochondrial electron transport chain (ETC) complex activities in TgHD and WT in the basal ganglia, frontal cortex and PBMCs (Fig. 4A,C,E). To evaluate whether mitochondrial protein levels were altered, despite normal ETC activity, as in human HD PBMCs (Askeland et al., 2018), western blot analyses were performed but revealed normal expression levels in the frontal cortex and PBMCs in WT and TgHD minipigs (Fig. S3).

The tissue-specific changes in mtDNA copy number in TgHD minipigs combined with the similar CS activities (Figs 2C and 4) could be indicative of different genotype-specific mitochondrial composition. We followed up on this possibility by first comparing cytochrome c oxidase (COX) activity in isolated mitochondria with that in tissue homogenates (Fig. S4). The results did not imply any difference in COX activity in isolated mitochondria. We then assessed coupled activities of ETC complex I+II and II+III. As shown in Fig. S5, the coupled activities confirmed the data obtained by measuring the single complex activities (Fig. 4).

### mHTT and HTT accumulate in an age- and tissue-specific manner in the TgHD minipig model

**Fig. 5.**
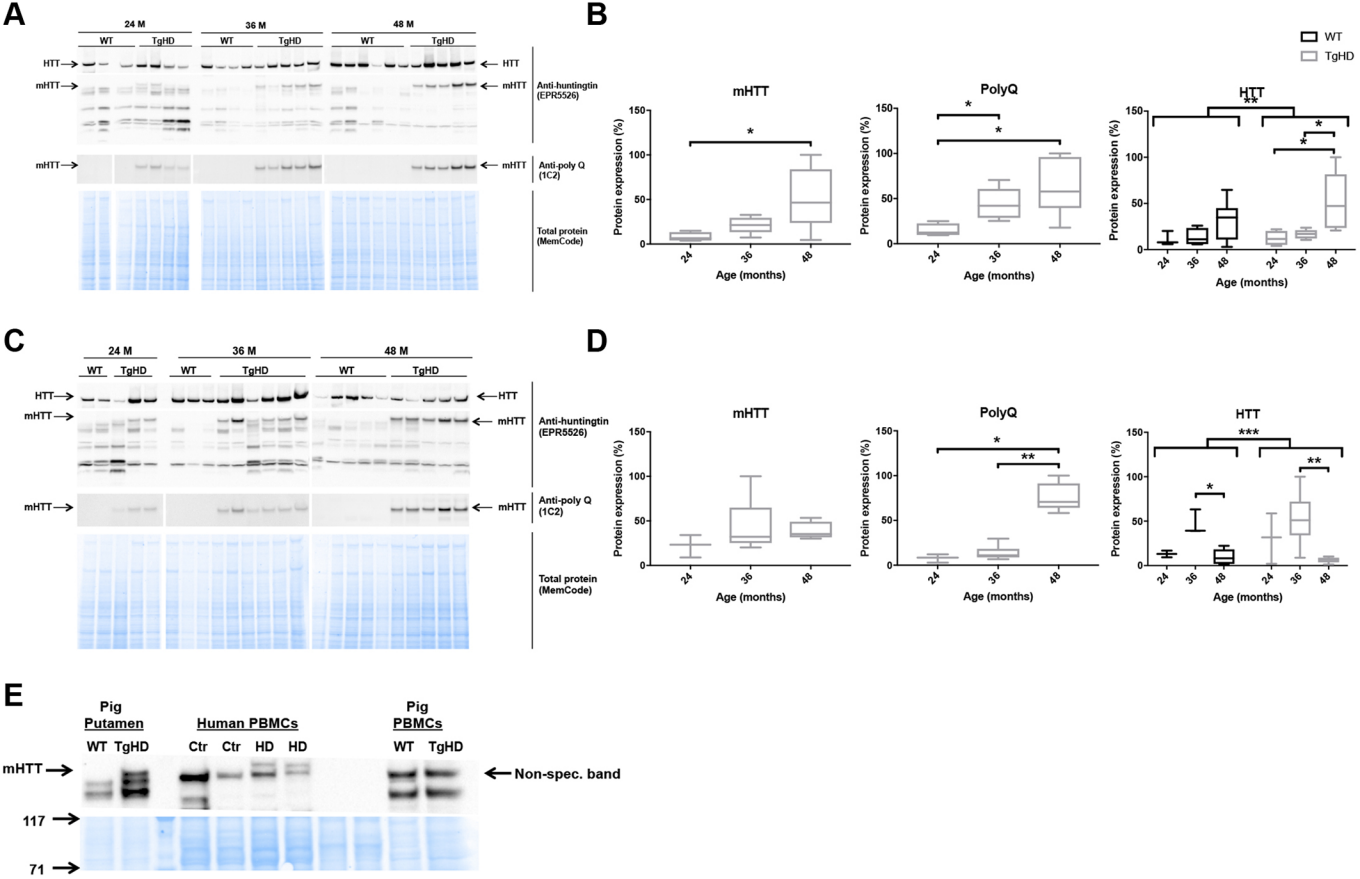
**TgHD minipig PBMCs do not express mHTT.** (A-D) Western blot analyses of individual minipigs and subsequent quantification revealed that mHTT and endogenous HTT were expressed in a tissue- and age-specific manner in the frontal cortex (A,B) (mHTT: TgHD 24 vs 48 months, *P*=0.04; PolyQ: TgHD 24 vs 48 months, *P*=0.02; TgHD 24 vs 36 months, *P*=0.02; HTT: TgHD 24 vs 48 months, *P*=0.03; TgHD 36 vs 48 months, *P*=0.02; ANOVA WT vs TgHD, *P*=0.004) and putamen (C,D) (PolyQ: TgHD 24 vs 48 months, *P*=0.04; TgHD 36 vs 48 months, *P*=0.004; HTT: WT 36 vs 48 months, *P*=0.04; TgHD 36 vs 48 months, *P*=0.009; ANOVA WT vs TgHD, *P*=0.001) in TgHD minipigs, and confirmed the absence of mHTT and PolyQ in WT animals. (E) mHTT was not expressed in minipig TgHD PBMCs. Representative western blot of mHTT in PBMCs from HD patients (HD) and controls (Ctr), and PBMCs from WT and TgHD minipigs, as indicated. Three distinct antibodies were used to identify mHTT and PolyQ fragments and endogenous HTT protein (see Materials and Methods). Mann–Whitney test and ANOVA, *P<0.05, **P<0.01, ***P<0.001. Sample sizes: (A,B) mHTT and PolyQ: TgHD 24 months, *n*=4; TgHD 36 months, *n*=5; TgHD 48 months, *n*=6; HTT: WT 24 months, *n*=3; WT 36 months, *n*=4; WT 48 months, *n*=6; TgHD 24 months, *n*=4; TgHD 36 months, *n*=5; TgHD 48 months, *n*=5; (C,D) mHTT and PolyQ: TgHD 24 months, *n*=3; TgHD 36 months, *n*=6; TgHD 48 months, *n*=5; HTT: WT 24 months, *n*=2; 36 months, *n*=3; 48 months, *n*=5; TgHD 24 months, *n*=3; 36 months: *n*=6, 48 months, *n*=5.

The results shown herein are indicative of subtle effects of the transgene on molecular biomarkers. To follow up on the putative impact of mHTT, we addressed the accumulation of mHTT and endogenous WT HTT protein in the tissues of interest. The transgene encoding the mHTT fragment was confirmed in the TgHD minipig, and was absent in WT minipigs (Fig. S1). As expected, in capillary electrophoresis, we observed a distinct peak representing 121 CAG/CAA repeats (Fig. S1B,C), which is different from the multiple peaks seen from repetitive CAG repeats that result in somatic expansions (Mollersen et al., 2012). To investigate the fate of endogenous HTT and transgene mHTT, we performed western blot analyses using specific antibodies against polyglutamine (PolyQ), mHTT and HTT. The N-terminal mHTT fragment accumulated with age in both the putamen and cortex (Fig. 5). The endogenous minipig HTT protein accumulated with age in the frontal cortex in both genotypes (Fig. 5A,B), but interestingly became depleted in the putamen at 48 months compared with 36 months (Fig. 5C,D). Importantly, the age-dependent accumulation of HTT was significantly different between TgHD and WT minipigs (ANOVA, *P*<0.001). Thus, the putamen from TgHD minipigs demonstrates a combination of reduced endogenous HTT protein with accumulation of mHTT fragment at the age of onset (48 months). PBMCs were recently shown to present important biomarkers for HD progression (Askeland et al., 2018). Cell-free extracts from PBMCs of minipigs at the affected age (48 months) were consequently analyzed for expression of mHTT. In contrast to human PBMCs, minipig TgHD PBMCs were found to express little or no mHTT fragments (Fig. 5E), thus explaining the relative inertness of these cells from the TgHD model.

In general, the relatively low impact of mHTT on the molecular parameters investigated so far is in coherence with the mild phenotype of the TgHD model. However, the most striking difference between human and the minipig model is the apparent lack of mHTT in TgHD PBMCs, which also makes PBMCs a less suitable biomaterial for studying disease progression in the TgHD model. Together, these results indicate that the mitochondrial capacity in the TgHD minipig model is not affected in the tested tissues before the age of 48 months.

## DISCUSSION

The TgHD minipig is a powerful HD model for use in intervention studies. Previous studies have demonstrated the toxic impact of mHTT protein on peripheral tissue and our study is the first to demonstrate neurological impairment, measured as decreased locomotor activity that initiates at an age of onset of 48 months. Hence, the TgHD minipig model could be an excellent model to capture and monitor biomarkers in response to interventions, in the pre-onset age up to 48 months.

The neurological phenotype in the TgHD model emerges subsequent to the reduced sperm count and motility (Macakova et al., 2016) in concordance with significantly diminished mitochondrial parameters (Krizova et al., 2017) and muscle ultrastructural alterations (M.R. and H.H., unpublished). The locomotor impairments that we identified here are partly in conflict with what was reported by Schuldenzucker and coworkers, who could not detect abnormal behavioral phenotype among female TgHD minipigs up to 48 months (Schuldenzucker et al., 2017). In our study, we did not investigate the impact of sex because of limited group size, but the change in the motor ability of TgHD boars compared with the WT controls appeared to be more pronounced than in TgHD sows compared with WT controls at the age of 48 months (data not shown), thereby providing a plausible explanation for the contradictory conclusions from the two studies. It should be noted, however, that the test repertoire was different in the two studies, except for the hurdle test, which in both studies did not show any statistical differences.

Prior to data analyses, we performed ANOVA to check for age association among the biomarkers for oxidative stress, DNA repair, genome integrity and mitochondrial function. We found no age association for any of these parameters and consequently pooled the samples into genotypes independent of age. It is a possibility that a larger cohort might indicate age-dependent effects for one or more of these parameters. However, currently the age correlation is too weak for these biomarkers to be used for monitoring. In contrast to the genomic and mitochondrial parameters, the expression of (m)HTT peptides in brain subregions varied with age. The N-terminal mHTT fragment accumulated strongly with increasing age in both the frontal cortex and basal ganglia, thus putatively exerting a toxic gain-of-effect in these brain subregions. The endogenous HTT protein displayed completely different tissue-dependent fates, in that the expression in the frontal cortex increased, whereas the expression in the basal ganglia became reduced at 48 months relative to 36 months of age. Interestingly, two-way ANOVA revealed a significant genotype difference with respect to age-dependent expression. Thus, the neurological phenotype could potentially be attributed to increased accumulation of toxic N-terminal mHTT fragments, or decreased expression of endogenous HTT protein, or a combination of both. Although HTT is essential for life, conditional knockout of gene function in adulthood is more tolerable. Wang and co-workers investigated the effect of knocking out *Htt* at various ages in mice, and found no neurological phenotype, but tendency to develop pancreatitis only at a young age (Wang et al., 2016a). This implies that the age-associated decline in endogenous HTT in the putamen is not responsible for the neurological phenotype in TgHD minipigs.

The PBMCs were relatively spared in the TgHD model, in contrast to what we have recently found in PBMCs from human patients. Time-resolved Förster resonance energy transfer (TR-FRET) as well as mRNA quantification methods have been used to confirm the expression of HTT and mHTT in immune cells from human patients (Björkqvist et al., 2008; Weiss et al., 2012), as well as in this study (Fig. 5E). The analogous mHTT fragments were also detected in lymphoid cells from the R6/2 mouse model (Träger et al., 2015), as analyzed by the TR-FRET method. Thus, our results imply that the minipig is different from humans and rodents in that minipig PBMCs do not accumulate mHTT to significant levels. This finding is in line with previously reported low mHTT levels in spleen from the TgHD minipig (Baxa et al., 2013). Consequentially, other types of peripheral tissue should be considered for monitoring biomarkers during interventions. Although we could not confirm mHTT expression in PBMCs, the 8-oxoG levels were elevated. Because medium components influence genomic integrity, it cannot be excluded that the increased 8-oxoG in TgHD PBMCs relates to different metabolite composition in the serum, as is the case for human patients (Underwood et al., 2006).

The TgHD minipig model is unique in that the PolyQ peptide is not encoded by repetitive CAG repeats. PolyQ repeats with CAG interruptions have been shown to be stable *in vivo* (Choudhry et al., 2001; Pearson et al., 1998), and this is supported by our findings (Fig. S1C). Previously, a similar strategy was used to generate a murine HD model expressing a stable PolyQ peptide (Gray et al., 2008), and thereby demonstrated that somatic instability is not required for neuropathology. Despite that several DNA repair functions have been demonstrated to catalyze somatic expansions, reports of the corresponding disease phenotype of the mouse mutants lacking these functions are still awaited. However, a mitochondrial superoxide quencher was used to delay onset of pathophysiology with a parallel reduction in somatic expansions (Budworth et al., 2015). Whether the therapeutic effects are caused by reduced oxidative stress or dampened CAG expansions – or a combination of both – remains to be tested. Although we did not detect evidence of general elevation of oxidative stress in the TgHD model, mitochondrial superoxide might still underlie neuropathology. As an example, the mtDNA mutator mouse that dies prematurely was not found to generate increased oxidative stress. In general, however, more refined measurement of mitochondrial subcompartments *in vivo* did reveal increased production of peroxide with age in this mouse model (Logan et al., 2014; Trifunovic et al., 2005). We identified lower mtDNA damage in the basal ganglia of the TgHD model, which suggests that mitochondrial peroxide stress should be lower rather than higher. In a recent study, we found lower mtDNA damage in human HD patients than in controls, which we believe is indicative of reduced mitochondrial activity (Askeland et al., 2018) and could be attributed to a similar bioenergetics dysfunction in human HD patients and TgHD minipigs.

The mtDNA copy number was differentially affected by mHTT expression in the frontal cortex and basal ganglia. A reduction in mtDNA copy number has been found in grade 3 postmortem HD brains (Siddiqui et al., 2012) and thereby supports the finding in the frontal cortex, although different brain regions were not compared. The reason for the increased mtDNA copy number in the basal ganglia is less obvious. It is a possibility that mitochondrial morphology differences could underlie the mtDNA:CS variations. Although we did not detect ETC activity differences, more experiments are needed to investigate mitochondrial fragmentation in the affected tissue (frontal cortex and basal ganglia). Using (1)H magnetic resonance spectroscopy, evidence was found of altered energy metabolism at pre-onset age in the TgHD minipig (Jozefovicova et al., 2016). DNA repair capacity, mtDNA integrity and metabolism are interdependent (Yuzefovych et al., 2013); therefore, the subtle alterations in the two former parameters found here might be an indication of altered metabolism in the TgHD minipig.

It is worth noting that the methylation marker 5-me(dC) was significantly increased in the frontal cortex in TgHD compared with WT. This might be an indication of an altered methylation state, reflected in the transcriptional dysregulation seen in HD (Benn et al., 2008). Epigenetic modifications, including DNA methylation and histone methylation, have been found in HD patients and animal models and show the emerging role of epigenetics in HD (Ferrante et al., 2003; Lee et al., 2013; Ng et al., 2013).

In conclusion, our characterization of the TgHD minipig model up to 48 months of age reveals the age of neuropathological onset. Further follow-up as the animals age will be crucial to map the pathological process in this novel HD model. It is interesting to note that HD biomarkers found in rodent models – such as oxidative stress, DNA damage and mitochondrial activity – were largely unaffected, suggesting that extrapolating data from evolutionarily distant models should be done with care. These findings also demonstrate the complexity of the disease and a need for additional biomarkers than those assessed here to monitor disease progression at the preclinical stage in this model, and potentially also in human patients to investigate future interventions.

## MATERIALS AND METHODS

### Minipig material and sample collection

Transgenic minipigs (*Sus scrofa domesticus*, Linnaeus) with the N-terminal part of human mutated huntingtin and their WT siblings (Baxa et al., 2013) were bred and studied in agreement with the Animal Care and Use Committee of the Institute of Animal Physiology and Genetics, under the Czech regulations and guidelines for animal welfare and with the approval of the Czech Academy of Sciences (protocol number 53/2015). After weaning, all piglets were genotyped as previously described (Baxa et al., 2013). At 24, 36 and 48 months of age, a cohort of the TgHD and WT minipigs was perfused under deep anesthesia with ice-cold PBS. The brain tissue was dissected and subregions such as the basal ganglia and frontal cortex were isolated and stored at −80°C after snap freezing in liquid nitrogen. In addition, the peripheral blood was collected with heparin-coated syringes. Within 1-2 h after collection, the whole fraction of intact lymphocytes was isolated from EDTA-blood by density centrifugation at 25°C, using HistopaqueR1077 Hybri-Max^TM^ (Sigma-Aldrich) following a standard protocol. Briefly, 6 ml blood was layered on top of 3 ml Histopaque and centrifuged at 800 ***g*** for 30 min at 25°C. The separated mononuclear band was carefully collected (∼1 ml), resuspended in 10 ml PBS (137 mM NaCl, 2.7 mM KCl, 4.3 mM Na_2_HPO_4_.12H_2_O, 1.5 mM KH_2_PO_4_) and centrifuged again at 800 ***g*** for 20 min. The pellet was washed twice in the same conditions. Finally, the dry pellet was stored at −80°C until use. For enzymatic protein and DNA analyses, samples from identical isolation of cells were used.

For isolation of the mitochondrial fraction, 5% homogenate (W/V) was prepared from the frozen tissue using Potter-Elvehjem homogenizer in KTEA medium (150 mM KCl, 50 mM Tris-HCl, 2 mM EDTA, pH 7.4, 0.2 μg/ml aprotininin) at 40°C. Mitochondria were sedimented by centrifugation of 600 g postnuclear supernatant at 10,000 ***g*** for 10 min at 4°C. The pellets were washed with KTEA medium and centrifuged again in the same conditions. Finally, the pellets were resuspended in KTEA medium to a protein concentration of ∼20 mg/ml. Measurement of mitochondrial enzymes was performed immediately after isolation. For western blotting and measurements of PDH activity, frozen aliquots were used.

### Human biomaterial

Use of human samples (PBMCs) was approved by the Ethical Committee of the General University Hospital in Prague [approved project COST LD15099 (2015-2017)] and the Czech-Norwegian Research Program (CZ09) 7F14308 (2014-2017). Written informed consent was obtained from patients as well as control subjects. The project was carried out in accordance with the principles expressed in the World Medical Association Declaration of Helsinki.

### DNA isolation and analysis

Total DNA was isolated using the DNeasy Blood and Tissue kit (Qiagen) following the manufacturer’s protocol with minor modifications. DNA purity and concentration was determined using a spectrophotometer (Epoch microplate spectrophotometer) and adjusted to correct concentrations for DNA integrity analysis (20 ng/µl for nDNA, 2 ng/µl for mtDNA). DNA integrity was assessed by the ability of a restriction enzyme to digest the DNA, using an in-house-developed quantitative PCR (qPCR)-based method as described previously (Wang et al., 2016b). Briefly, the DNA template was incubated with the *Taq*αI restriction enzyme, and primers flanking the *Taq*αI sites in the mtDNA and nDNA were subsequently used to amplify a PCR product. The ability to amplify across the restriction site is proportional to the amount of mtDNA and nDNA damage. Primer sequences are provided in Table S1.

CAG sizing was performed by PCR using primers (Table S1) with a fluorescent tag designed to flank the region of the CAG repeats. PCR products were run on a 1.2% agarose gel, and the resulting band was cut out and separated by capillary electrophoresis. The lengths of the PCR products were determined by fragment analysis on an Applied Biosystems 3130 Genetic Analyzer. Data analysis was performed by GeneMapper^®^ software V2.6.4.

mtDNA copy number analysis was carried by qPCR amplification of a nuclear (*HBB*) and a mitochondrial (*MT-RNR1*) amplicon. Relative mtDNA content was calculated based on the ratio of mtDNA product over nuclear DNA products, using standard curves.

DNA base modifications were determined using liquid chromatography-mass spectrometry/mass spectrometry (LC-MS/MS) at the PROMEC proteomics and metabolomics core facility [Norwegian University of Science and Technology (NTNU), Trondheim], as described previously (Askeland et al., 2018).

The mutation frequency in mtDNA was determined as described previously (Wang et al., 2015). Briefly, total DNA was mixed with S1 nuclease (Qiagen; 10 U) for 15 min at 37°C and later digested using *Taq*αI restriction enzyme (New England Biolabs; 100 U, 65°C for 15 min). The resistant mutated *Taq*αI restriction sites were quantified by qPCR using the specific primers listed in Table S1. To ensure complete digestion, an additional *Taq*αI treatment (100 U; 65°C for 15 min) was performed on the digested DNA samples prior to qPCR. Mutation frequency was calculated as (2exp(CT^TaqI^−CT^NT^)×4)^−1^ per nucleotide. Primer sequences are provided in Table S1.

### Western blot analyses

Cell lysates of PBMC pellets were obtained using RIPA buffer (150 mM NaCl, 5 mM EDTA pH 8, 0.05% NP-40, 1% sodium deoxycholate, 0.1% SDS, 1% Triton X-100, 50 mM Tris-HCl pH 7.4, inhibitors of phosphatases and proteases) and protein concentration determined. Frozen tissues were homogenized in liquid nitrogen using a mortar and lysed in RIPA buffer, sonicated for 15 min at 0°C and centrifuged at 15,000 ***g*** for 15 min at 4°C. The concentration of protein was determined, and same amount of total protein was loaded onto a 3-8% Tris-acetate gel (EA03758, LifeTech). After electrophoresis, proteins were transferred to nitrocellulose membrane and stained with MemCode (LifeTech) to visualize total protein.

The membranes were blocked in blocking buffer and incubated with primary antibodies (see below) overnight at 4°C. Secondary detection was carried out with horseradish peroxidase (HRP)-conjugated secondary antibodies (see below). The antibody-bound proteins were visualized with the Super Signal West Femto Maximum Sensitivity Substrate (Thermo Fisher Scientific) using a Syngene Imaging System, and the intensity of the signal was quantified using Quantity One 1-D Analysis Software (Bio-Rad).

For huntingtin detection, anti-HTT antibody (EPR5526, Abcam, 1:2000) and anti-PolyQ (clone 5TF1-1C2, MAB1574, Millipore, 1:2000) were incubated overnight at 4°C. Corresponding secondary antibodies conjugated to HRP (anti-mouse, 711-035-152, Jackson ImmunoResearch, 1:10,000 or anti-rabbit, 711-035-152, Jackson ImmunoResearch, 1:10,000) were used. The signal was detected by chemiluminescence (ECL, 28980926, APCzech) and proteins were detected by a ChemiDoc XRS+System (Bio-Rad). MemCode (1858784, Thermo Fisher Scientific) total protein staining was used for normalization of loading. For details of antibodies used in western blotting, see Table S2.

### Mitochondrial enzyme activities

The activities of the respiratory chain complexes NADH:ubiquinone oxidoreductase (NQR, complex I), succinate:CoQ reductase (SQR, complex II), ubiquinol:cytochrome c oxidoreductase (QCCR, complex III), cytochrome c oxidase (COX, complex IV), NADH:cytochrome c reductase (NCCR, complex I+III), succinate:cytochrome c reductase (SCCR, complex II+III) were measured spectrophotometrically at 37°C in tissue homogenate and/or isolated mitochondria (Rustin et al., 1994) and CS according to Srere (1969). Protein concentration was measured according to Lowry et al. (1951). PDH activity was determined by measuring ^14^CO_2_ production by decarboxylation from [1-14C]pyruvate according to Cahova et al. (2015). Analysis of respiratory chain complexes I, II, IV and CS in PBMCs has previously been described in detail (Askeland et al., 2018).

### DNA repair activity

DNA glycosylase activity in cell-free nuclear protein extracts was performed as described previously (Klungland et al., 1999). Briefly, nuclear extracts were prepared by osmotic extraction, and 0.5-10 µg was added to a reaction mixture containing 50 mM MOPS pH7.5, 1 mM EDTA, 5% glycerol, 1 mM DTT and 1 fmol ^32^P-endlabeled 8-oxoG-containing duplex and incubated for 2 h at 37°C. The reaction was terminated by addition of formamide/loading dye mixture, and denaturing at 85°C for 3 min. The incised product was separated from substrate oligonucleotide by electrophoresis on a 15% urea-PAGE gel. The gels were dried and subjected to phosphoimaging using a Typhoon 9410 Variable Mode Imager. The DNA glycosylase activity was calculated based on the ratio of incised oligonucleotide (product) over incised oligonucleotide+remaining substrate, and related to protein amount. The calculated activity was normalized to that of the average of controls, and presented with box plots displaying the 25th and 75th percentiles.

### Lipid peroxidation assay

The lipid peroxidation assay was carried out using a TBARS assay kit (Cayman Chemical). Tissue homogenates were prepared from small amounts of tissue (∼25 mg) using RIPA buffer. The assay was performed according to the manufacturer's protocol using fluorometric standards and a Wallac VICTOR2™ 1420 multilabel counter. The results were presented as MDA formed (A.U.), according to the manufacturer.

### Motor control assessment

TgHD boars (*n*=2) and their WT controls (*n*=2) together with TgHD sows (*n*=4) and their WT controls (*n*=4) at 48 months of age were used for motor assessment. The animals were starved and a biscuit treat was used to stimulate the animals to perform the test. The following tests were performed: balance beam, hurdle, seesaw, gait and tunnel test.

The balance beam consisted of 2.5 m long inclined plane, 3.0 m long beam and extended plane (1.15×1.3 m). The animal is expected to step up to an inclined plane, cross the beam, turn back in the extended part and return back down. Scoring was as follows: 5 points for passing across the whole beam, turning in the extended part and going back down; 4 points for passing across the whole beam, not turning in the extended part and retreating; 3 points for stepping on (any part of) the beam; 2 points for stepping on the inclined plane with all four legs; 1 point for stepping on the inclined plane with two (fore)legs; 0 points if the animal refused to perform the test.

In the hurdle test, the animals were expected to pass the hurdle (height, 15 cm; width, 100 cm). Scoring was as follows: 5 points for passing across the hurdle without touching; 4 points for passing across the hurdle with touching of one leg; 3 points for passing across the hurdle with touching of two legs; 2 points for passing across the hurdle with touching of three legs; 1 point for passing across the hurdle with touching of four legs; 0 points if the animal refused to perform the test.

During the seesaw test, the pig was expected to pass over the seesaw (length, 3.0 m; width, 0.4 m). Scoring was as follows: 5 points for passing across the whole seesaw; 4 points for passing to the equilibrium position and retreating; 3 points for stepping onto and walking on the seesaw with all four legs (the equilibrium position is not reached); 2 points for stepping on the seesaw and retreating; 1 point for stepping on the seesaw with only two (fore)legs; 0 points if the animal refused to perform the test.

During the gait test, changes (difficulties) in walking were observed by walking the animal on a dry, straight floor. Scoring was as follows: 5 points for no visible gait problem and fluent walking; 4 points for slightly uneven weight bearing on one or more legs; 3 points for obvious deviation in weight bearing on one or more legs, with clear difficulties in walking; 2 points for lowering of hind quarters close to the ground, placement of hind legs under the body; 1 point for sliding the legs out of the symmetry; 0 points if the pig was unable to move and perform the test.

During the tunnel test, three biscuits were placed in different spots in the tunnel (diameter, 0.5 m; length, 1.5 m). The animal was expected to eat all the biscuits. Scoring was as follows: 5 points if three biscuits were eaten; 4 points if two biscuits were eaten; 3 points if only one biscuit was eaten; 2 points for entering the tunnel but not eating any biscuits; 1 point for showing no fear to enter but not entering the tunnel; 0 points if the animal refused to perform the test.

The tests were genotype blinded, meaning that the evaluators did not know the genotype of the animal.

### Statistics

All statistics were carried out in GraphPad Prism. Calculation of statistical significance was performed using Student's *t*-test, Mann–Whitney or two-way ANOVA, as stated. We used the Holm–Sidak method for adjustment for multiple comparisons, where applicable.

## Supplementary Material

Supplementary information

First Person interview
