# Supplementary material for: A transgenic minipig model of Huntington's disease shows early signs of behavioral and molecular pathologies
Source: Dis Model Mech. 2018 Oct 24;11(10):dmm035949. doi: 10.1242/dmm.035949 (PMC6215428; doi:10.1242/dmm.035949)
Supplement: Supplementary information [file dmm-11-035949-s1.pdf]

FIRST PERSON

First person – Georgina Askeland

First Person is a series of interviews with the first authors of a selection of papers published in Disease Models & Mechanisms, helping early-career researchers promote themselves alongside their papers. Georgina Askeland is first author on 'A transgenic minipig model of Huntington's disease shows early signs of behavioral and molecular pathologies', published in DMM. Georgina conducted the work in this article while a PhD student in the lab of Prof. Lars Eide at the University of Oslo, Norway, investigating neurodegenerative disease with a focus on mitochondrial dysfunction and DNA damage/ repair. She is now an Innovation Consultant/Project Developer at Innovayt AS, Oslo, Norway.

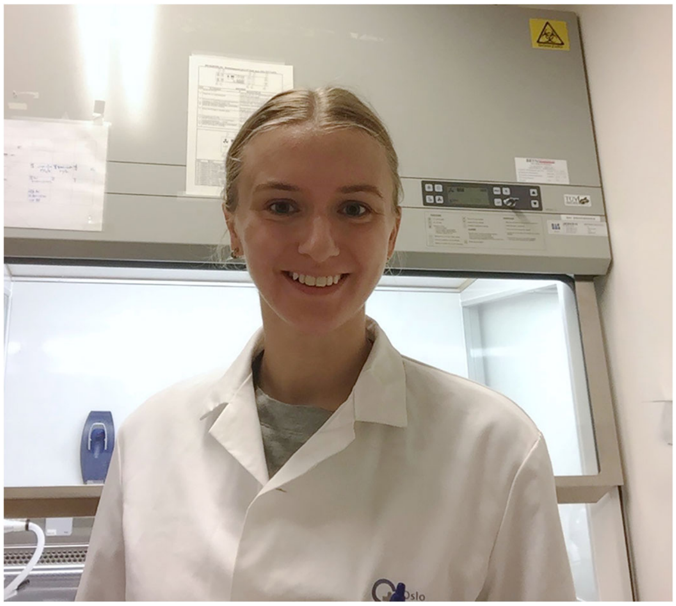

Georgina Askeland

How would you explain the main findings of your paper to non-scientific family and friends?

Huntington's disease (HD) is a rare but devastating genetic disease that affects the brain and causes movement difficulties and dementia, among other symptoms. Currently, there are no treatments that can slow or prevent the disease. Scientists often use animal models to understand how a disease works and to find new drugs to treat a disease. Mice are the most common models for human disease as they are small, easy to breed and reach adulthood quickly. However, for diseases that affect the aging brain, pigs can be a better model due to their larger, more complex brains and longer lifespan. In our study we showed that our newly developed 'minipig' model of HD begins to show symptoms of neurodegeneration at 48 months, suggesting that this model reflects the human disease. We also found cellular changes in various brain regions in the model affecting mitochondrial DNA and DNA repair, which may result from neurodegeneration.

"[…] we hope that this pig model can help increase the translatability of treatments in development for their use in humans."

What are the potential implications of these results for your field of research?

This minipig model of HD has the potential to improve preclinical testing for new therapeutics for this currently untreatable disease. Its larger brain size and anatomical similarities to humans means that drug delivery to the brain can be monitored, for example, using MRI. A number of promising treatments tested in mouse models have previously failed when tested in humans and we hope that this pig model can help increase the translatability of treatments in development for their use in humans.

What are the main advantages and drawbacks of the model system you have used as it relates to the disease you are investigating?

The main advantages of this model are its larger brain size, longer lifespan and greater anatomical similarities to humans compared to mice. For neurodegenerative diseases, these characteristics are particularly important to be able to properly model changes to the brain over time. However, a longer lifespan means that the research takes longer than it would with mice and more research is required to fully characterize this model as the animals age.

What changes do you think could improve the professional lives of early-career scientists?

Teaching early-career scientists the translatability of their skills and the opportunities within commercialization and entrepreneurship is key. Highly qualified researchers have a wealth of experience and knowledge that can be used to develop new biotechnologies and medical innovations. This can benefit the individual scientist by broadening their understanding of opportunities, while simultaneously driving healthcare improvements for society.

"Teaching early-career scientists the translatability of their skills and the opportunities within commercialization and entrepreneurship is key."

What's next for you?

I will be starting a new position shortly as an innovation consultant/ project developer at a leading innovation consultancy company in the Nordics. This will provide a great opportunity to use my skills from my research career to help companies explore and develop their innovative projects.

Georgina Askeland's contact details: Innovayt AS, Forskningsparken, Gaustadaléen 21, 0349 Oslo, Norway.
E-mail: georgina.askeland@me.com

Disease Models & Mechanisms (2018): doi:10.1242/dmm.035949: Supplementary information

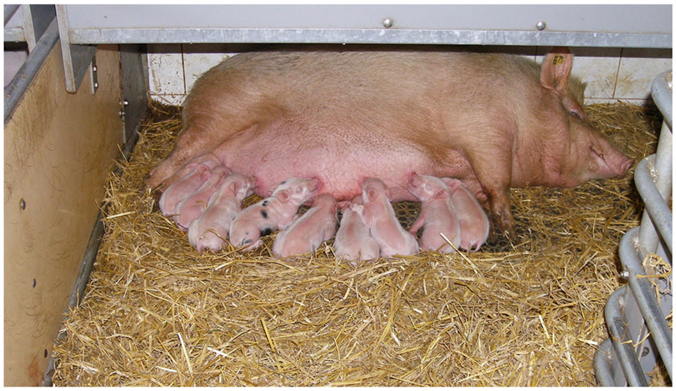

Newborn litter of HD model minipigs.

Reference

Askeland, G., Rodinova, M., Štufková, H., Dosoudilova, Z., Baxa, M., Smatlikova, P., Bohuslavova, B., Klempir, J., Nguyen, T. D., Kuśnierczyk, A., Bjørås, M., Klungland, A., Hansikova, H., Ellederova, Z. and Eide, L. (2018). A transgenic minipig model of Huntington's disease shows early signs of behavioral and molecular pathologies. *Dis. Model. Mech.* **11**, dmm035949.
